# Distractor suppression leads to reduced flanker interference

**DOI:** 10.3758/s13414-020-02159-z

**Published:** 2020-12-02

**Authors:** Yavor Ivanov, Jan Theeuwes

**Affiliations:** 1 Department of Experimental and Applied Psychology, Vrije Universiteit Amsterdam, Van der Boechorststraat 7, 1081 BT Amsterdam, The Netherlands; 2 Institute of Brain and Behavior Amsterdam (iBBA), Amsterdam, The Netherlands

**Keywords:** Attention, Statistical learning, Distractor suppression, Top-down/bottom-up, Habituation

## Abstract

Recent studies using the additional singleton paradigm have shown that regularities in distractor locations can cause biases in the spatial priority map, such that attentional capture by salient singletons is reduced for locations that are likely to contain distractors. It has been suggested that this type of suppression is proactive (i.e., occurring before display onset). The current study replicated the original findings using an online version of the task. To further assess the suppression of high-probability locations, we employed a congruence manipulation similar to the traditional flanker effect, where distractors could be either congruent or incongruent with the response to the target. Experiment 1 shows that through statistical learning distractor suppression reduces the interference from incongruent distractors, as participants made less errors in high-probability versus low-probability conditions. In Experiment 2, participants were forced to search for a specific target feature (the so-called feature-search mode), which is assumed to allow participants to ignore distractors in a top-down manner. Yet even when this “top-down” search mode was employed, there was still a congruence effect when the distractor singleton was presented at the low-probability but not at the high-probability location. The absence, but not reversal, of a congruence effect at the high-probability location also further indicates that this distractor suppression mechanism is proactive. The results indicate that regardless of the search mode used, there is suppression of the high-probability location indicating that this location competes less for attention within the spatial priority map than all other locations.

To interact with our immediate environment, it is crucial that we focus on information that is relevant to us and avoid distraction by irrelevant salient information. Selective attention makes it possible to enhance the processing of particular elements in a visual display (C. W. Eriksen & Hoffman, 1974), while filtering out irrelevant information (Broadbent, 1958). Most theories adhere the position that selective attention is the result of an interaction between the goals of the observer (top-down control; goal-driven; Egeth & Yantis, 1997) and the properties of the stimuli in the environment (bottom-up; stimulus-driven; Theeuwes, 2010).

Even though this top-down and bottom-up dichotomy is well established, recently it was argued that a third source of attentional control, dubbed “selection history” has a strong effect on attentional control (Awh, Belopolsky, & Theeuwes, 2012; Failing & Theeuwes, 2018; Theeuwes, 2018, 2019). One of the most well-known examples of how previous selection episodes (cf. selection history) affect attentional selection is research known as “contextual cueing” in which it is shown that search for a target is facilitated when it appears in a visual layout that was previously searched relative to visual layouts that were never seen before (Chun & Jiang, 1998, 1999, 2003; Jiang & Chun, 2001). The notion is that participants learn statistical regularities present in the display, which in turn makes them faster in finding targets appearing in previously searched displays relative to new displays.

Recent research has shown that people can learn regularities not only about the target location but also about distractor locations (Failing, Wang, & Theeuwes, 2019; Wang & Theeuwes, 2018a, b, c, Wang and Theeuwes, 2020a, b; Wang, Samara, & Theeuwes, 2019b). These studies employed the additional singleton task (Theeuwes, 1992) in which observers have to search for a unique shape singleton while a unique and salient color singleton is simultaneously present. Instead of presenting the singleton distractor evenly across all positions in the display, Wang and Theeuwes presented the distractor singleton much more often in one location than in all other locations. The results showed that a color singleton distractor at a high-probability location caused less attentional capture than when this color distractor was presented at a low-probability location. In other words, the distracting effect of the color singleton was attenuated (see Ferrante et al., 2018, for similar results).

Wang and Theeuwes (2018a) suggested that capture was reduced because the location that contained a distractor more frequently was suppressed relative to other locations. Specifically, it was argued that learning the regularities of the environment would lead to plastic changes in the spatial priority map such that an object positioned at that suppressed location would compete less for attention than objects presented at other locations in the visual field. Statistical learning is assumed to modify the weights for each item in a display as assigned in the priority maps, which in turn regulate the distribution of spatial attention in a dynamic fashion (Fecteau & Munoz, 2006; Godijn & Theeuwes, 2002; Itti & Koch, 2001; Theeuwes, 2019).

A recent electroencephalography (EEG) study by Wang et al. (2019a, b) provided evidence that the high-probability location is proactively suppressed, implying that before display onset, the high-probability location is already suppressed relative to all other locations (see also Ferrante et al., 2018, for similar arguments). Because Wang et al. (2019a, b) found that there was prestimulus (i.e., before display onset) enhanced parieto-occipital alpha power contralateral to the high-probability location, it was concluded that this suppression had to be proactive. This finding is consistent with results obtained in an eye-tracking study (Wang, Samara, et al., 2019b), which showed that fewer saccades landed at the distractor when it was presented at the high-probability location than at a low-probability location.

This type of proactive suppression can be contrasted with reactive suppression that operates later in time and only after attention was first directed to the suppressed location (Won, Kosoyan, & Geng, 2019). In other words, attention is first captured towards the high-probability distractor, but due to reactive suppression it is rapidly disengaged away from its location. This notion of rapid disengagement is similar to Moher and Egeth’s (2012) “search and destroy” hypothesis. They showed that participants that were instructed to suppress an object with a particular color could only do so after attending to the location of the to-be-ignored color.

The present study was designed to provide further and different evidence for the suppression of the high-probability location relative to the low-probability location. To that end we made use of a technique reminiscent of the classic B. A. Eriksen and Eriksen (1974; C. W. Eriksen & Hoffman, 1972, 1973; C. W. Eriksen & Schultz, 1979) flanker effect. In a typical flanker experiment, participants have to identify a target letter appearing at a particular location. Typically, two target letters are linked to the same response. For example, participants have to press one button if the target is an *H* or an *M* and to press another button if the target is an *A* or a *U*. In the classic flanker task, the target is surrounded on either side by task-irrelevant flanker letters that can be mapped to the same or a different response as the target. Participants have to respond to the target and ignore the flankers. The typical result is that response times are affected by the identity of the flanking stimuli: RTs are longer when the flankers are response incongruent than when the flankers are congruent with the response to the centrally presented target letter.

In the current study, we employed the classic additional singleton task (Theeuwes, 1991, 1992), in which participants search for a shape singleton while ignoring a color distractor singleton. Typically, in tasks like these, participants respond to the orientation of the line segment (e.g., press right when vertical; press left when horizontal) that is placed within the target singleton. In the current study, we changed the response requirements: participants had to press a button with their left-hand index finger when the left side of the shape singleton was filled and press another button with the right-hand index finger when the right side of the shape singleton was filled (see Fig. 1). These changes ensured that the responses were compatible with what was displayed (left fill = left response, right fill = right response). Critically, all other elements in the display also contained filled sides including the color singleton distractor. This creates a condition comparable to the B. A. Eriksen and Eriksen (1974) flanker task, such that the side within the distractor singleton can be congruent with the response to the target singleton (both left side or both right side) or can be incongruent (one left and one right side; see also Theeuwes, 1995, who employed a similar technique).

**Fig. 1:**
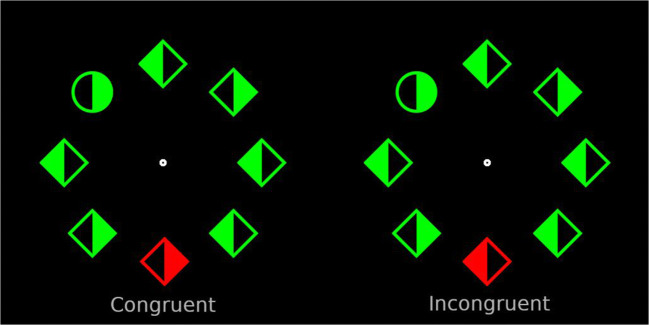
Example search displays from Experiment 1. The display to the left represents the congruent condition; the display to the right represents the incongruent condition. Stimuli are not displayed to size

Given this congruence manipulation there are in principle three possible outcomes. First, if the singleton distractor captures attention, the orientation of the distractor is processed and will result in a congruence effect. Second, if the location of the distractor is proactively suppressed (see Wang et al., 2019a, b), the orientation of the element inside the distractor will not affect responding (no congruence effect). The reasoning here is that if there is no attention going to the distractor (no or little attentional capture), the orientation of the distractor is not processed and therefore does not become available for response. Because the orientation of the distractor is not available, it cannot result in a congruence effect (see Theeuwes & Van der Burg, 2011; Theeuwes, Van der Burg, & Belopolsky, 2008). Finally, it is possible that suppression is reactive indicating that attention is captured by the salient distractor, but then immediately suppressed and disengaged from its location. This may in principle result in a reverse congruence effect. Because the identity of the orientation of the distractor is processed and then suppressed, responding to an orientation that is congruent with that of the target may be slower and more inaccurate (because it is suppressed) than when it is incongruent.

The current study was modeled after the experiments in Wang and Theeuwes (2018a, b). Participants searched for a salient shape singleton (i.e., a diamond between circles or a circle between diamonds) while ignoring a colored distractor singleton. We presented the colored distractor singleton systematically more often in one location (high-probability location) than in all other locations (low-probability locations). As in Wang and Theeuwes (2018a, b), we expected less capture by the salient singleton when it is presented at the high-probability relative to the low-probability locations. The question we addressed is whether the congruence effect is affected by this manipulation as well. If the high-probability location is indeed suppressed we expect that the congruence effect is smaller for distractors presented at the high-probability location relative to ones presented at the low-probability location. If distractor suppression is reactive, we expect that the congruence effect at the high-probability location may even reverse its direction. Thus, finding that distractor suppression reduces the congruence effect would provide additional evidence for feature-blind suppression of the high-probability location.

## Experiment 1

In Experiment 1, we employed the same paradigm as Wang and Theeuwes (2018a, b), in which participants searched for a salient shape singleton (i.e., a diamond between circles or a circle between diamonds) while ignoring a colored distractor singleton. Because the target was a unique singleton in the shape dimension, participants needed to employ the so-called singleton-detection mode (Bacon & Egeth, 1994), in which there is also strong distractor interference as the distractor is the only feature singleton on the display other than the target. As the experiment is similar to Wang and Theeuwes (2018a, b) for the high-probability location, we expect to find (1) a reduction in the amount of attentional capture by distractors, and (2) less efficient selection of the target when presented at the high-probability location. In addition, if the high-probability location is proactively suppressed, we predict a reduced congruence effect between target and distractor (i.e., slower RTs and reduced accuracy in the incongruent relative to the congruent condition) when the distractor is presented at the high-probability location relative to the low-probability location. If, however, suppression is reactive, then we expect to find a reversed congruence effect (i.e., slower RTs and reduced accuracy in the congruent relative to the incongruent condition). Because the identity of the orientation of the distractor is processed and then suppressed, the response associated with that orientation may become suppressed as well, resulting in slower responding when the orientation of the distractor is congruent with that of the target, relative to when it is incongruent. For example, Hübner and Töbel (2019) found reversed flanker effects in a task in which flankers were presented ahead of time to the centrally presented target, forcing the processing of the flankers before the target, creating a situation comparable with the reactive suppression mechanism described here.

### Method

The study was approved by the Ethical Review Committee of the Faculty of Behavioral and Movement Sciences of Vrije Universiteit Amsterdam.

#### Participants

Participant recruitment was done through Prolific (www.prolific.co). Sixty-four participants signed in and completed the task online. Fifteen participants dropped out before finishing the study, and their data were removed from the analysis. One participant was removed due to performance below chance level, and one due to abnormally long response times (>3,000 ms). Thus, the reported analyses are based on a sample of 47 participants (23 females; *M*_age_ = 28.3 years). Sample size was justified with a power analysis based on the main effect of distractor location as reported in Wang and Theeuwes (2018a). With partial η^2^ = *.*85 and alpha = 5%, power for the critical effect was >.99. For the congruence effect, we based our power analysis on the results of Theeuwes and Van der Burg (2011) as they used a similar congruence manipulation in the additional-singleton paradigm. With an effect size (*d*_*z*_) of approximately 2.16 and alpha = 5%, our current sample size achieves >.99 power for the critical effect. All participants reported normal to corrected-to-normal (color) vision. Before the experiment, participants provided informed consent via button-press.

#### Apparatus and stimuli

The experiment was designed using OpenSesame v.3.3.1 (Mathôt, Schreij & Theeuwes, 2012) with the OSWeb v.1.3.8 extension for online experiments. Participants used their own desktop or laptop computers to do the task; therefore, the experimental setup varied between sessions. The values presented in this section were obtained by running the study in Google Chrome on a 15.6-inch screen with a resolution of 1,920 × 1,080 px.

As illustrated in Fig. 1, the visual search array consisted of eight discrete stimuli with different shapes: either a circle with a radius of 50 px among diamonds subtending 85 × 85 px, or vice versa, displayed on a black background. Each shape had either a red or green color. Stimuli were centered around a fixation dot with a radius of 8 px. Each stimulus was filled with color either to the left or to the right side of its vertical axis, and was empty (i.e., background color) on the opposite side.

#### Procedure and design

Each trial began with the presentation of the fixation dot for a random duration between 500 and 750 ms. Then the search display was shown for 3,000 ms or until response. Participants had to search for a uniquely shaped target (e.g., one circle among diamonds, or vice versa), and indicate whether its colored area is to its left or right by pressing the *Z* or *M* keys, respectively. Participants were instructed to keep their left-hand and right-hand index fingers above these keys at all times while performing the task.

The target was present on each trial, and its shape was randomly determined. A uniquely colored distractor singleton was shown on 66% of the trials. Crucially, on half of those trials the side at which the distractor was filled in was congruent with the target stimulus (i.e., right-right), and on the other half of the trials it was incongruent (i.e., right-left; see Fig. 1). Additionally, as in Wang and Theeuwes (2018a, b), one location was associated with a high probability of containing the distractor singleton (i.e., 66% of distractor-singleton-present trials; 44% of all trials); all other locations had a low probability (33%) of containing the distractor singleton. The high-probability location was randomly assigned between participants. In conditions where a distractor singleton was absent, the location of the target was randomly determined on each trial. Participants completed 18 practice trials in which the distractor singleton was equally likely to appear in every location. If their accuracy during practice was below 55%, they were reminded of the instructions and had to perform an additional 18 practice trials until their performance improved. The experiment had three blocks each, consisting of 144 trials. After the experiment was done, participants were debriefed about the purpose of the study.

### Results

Trials in which response times (RTs) were below or above a 2.5 standard deviations cutoff point (within-participants) were excluded from analyses (2.2% of all trials).

#### Attentional capture and distractor suppression

We first determined whether we replicated the findings of Wang and Theeuwes (2018a, b) who showed reduced attentional capture when the singleton distractor is presented at the high-probability versus the low-probability location. Also, we included a factor of distractor location repetition in our analysis to assess whether trial-to-trial repetitions (which, due to the design, are much more frequent for the high-probability location) would contribute to the effect.

**Fig. 2:**
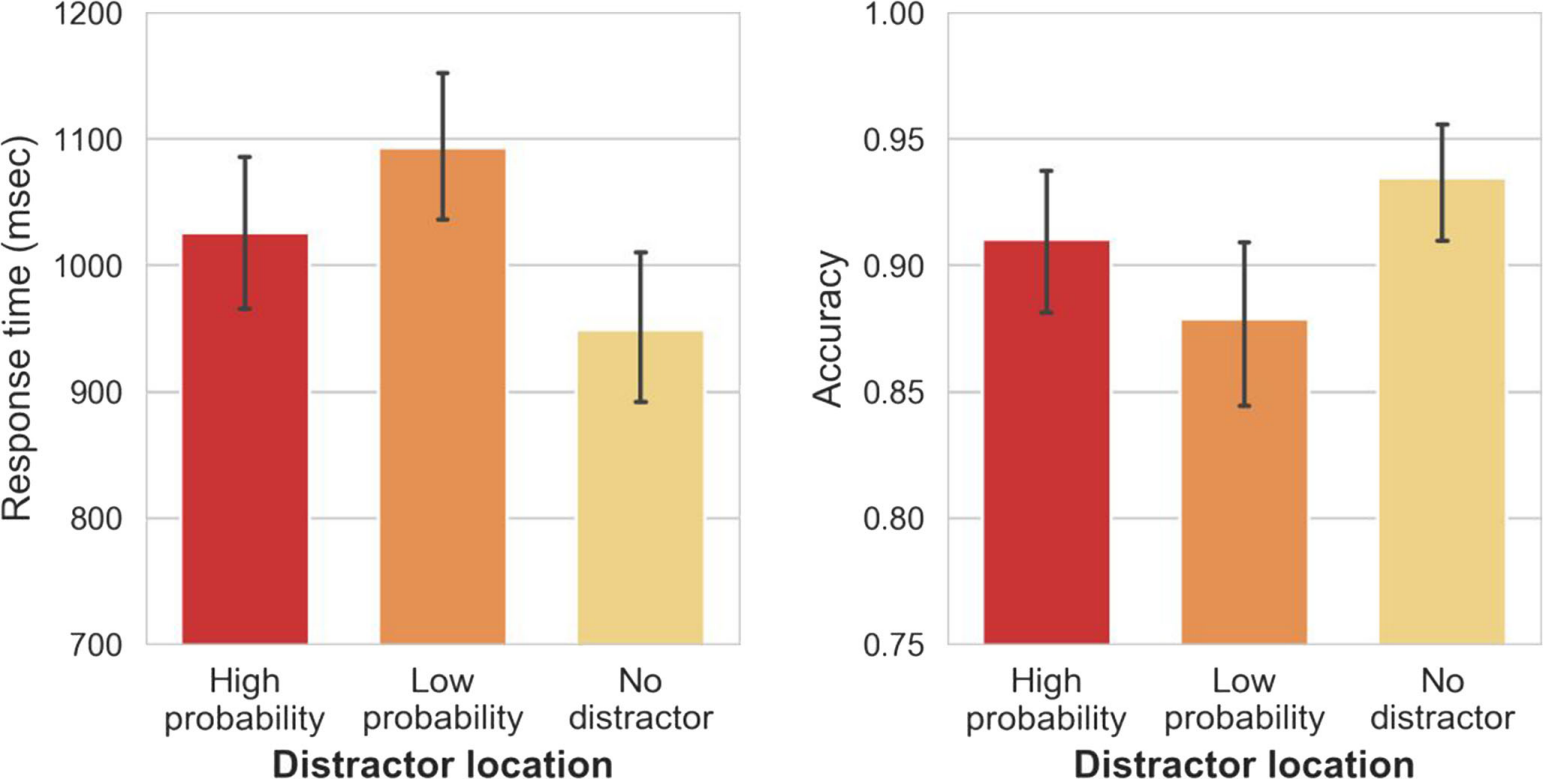
The mean response times (RTs; left panel) and mean accuracy (right panel) between different distractor conditions and repetition conditions in Experiment 1. Error bars represent 95% confidence interval (CI)

Mean RTs are shown in Fig. 2 (right panel) and were analyzed using a two-way repeated-measures (RM) analysis of variance (ANOVA) on mean RT, with distractor location (no distractor vs. high-probability vs. low-probability) and repetition (yes vs. no) as factors. Statistics are reported with the Greenhouse–Geisser sphericity correction where necessary. Singleton location had a significant main effect on RTs, *F*(1.62, 74.64) = 98.17, *p <* .001, η_p_^2^ = .68. Repetition did not have a main effect, *F*(1, 46) = 2.95, *p =* .09, η_p_^2^ *=* .06. A Bayesian analysis revealed that the data are 5.52 (BF_01_) times more likely to occur under a null model than a model with repetition as a main effect, providing substantial evidence that the effect of location was indeed driven by statistical regularities in the display and not merely by trial-to-trial repetitions. Post hoc comparisons show that responses to the target in the no-distractor condition were on average 76-ms faster than in the high-probability condition, *t*(46) = 7.41, *p*_*bonf.*_ *<* .001, *d =* 1.08; and 145-ms faster than in the low-probability condition, *t*(46) = 14.004, *p*_*bonf.*_ *<* .001, *d =* 2.04. Crucially, responses in high-probability locations were 68-ms faster than in low-probability locations, *t*(46) = 6.59, *p*_*bonf.*_ < .001, *d =* 0.96, suggesting that relative to the low-probability location attentional capture was attenuated for the high-probability location. These results are completely consistent with the findings of Wang & Theeuwes (2018a).

Mean accuracy is shown in Fig. 2 (right panel), and was analyzed using a two-way RM-ANOVA, with distractor location (no distractor vs. high-probability vs. low-probability) and repetition (yes vs. no) as factors. Distractor location had a main effect on accuracy, *F*(1.37, 63.21) = 19.76, *p* < .001, η_p_^2^ *=* .30. Repetitions had no effect on accuracy, *F*(1, 46) = 2.18, *p* = .147, η_p_^2^ = .04. Post hoc comparisons show that mean accuracy in the no-distractor condition was 2% higher than in the high-probability condition but this difference was not reliable, *t*(46) = 2.26, *p*_*bonf.*_ *=* .08, *d =* 0.33. However, a Bayesian analysis strongly suggests that this difference may actually be significant (*BF*_*10*_ > 43,000). Accuracy in the no-distractor condition was 5% higher than in the low-probability condition, *t*(46) = 6.21, *p*_*bonf.*_ < .001, *d =* 0.91. In high-probability locations, mean accuracy was 3% higher than in low-probability locations, *t*(46) = 3.95, *p*_*bonf.*_ *<* .001, *d =* 0.58, or in other words, participants made fewer errors when the distractor appeared in the high-probability location versus when it appeared at any of the other locations.

**Fig. 3:**
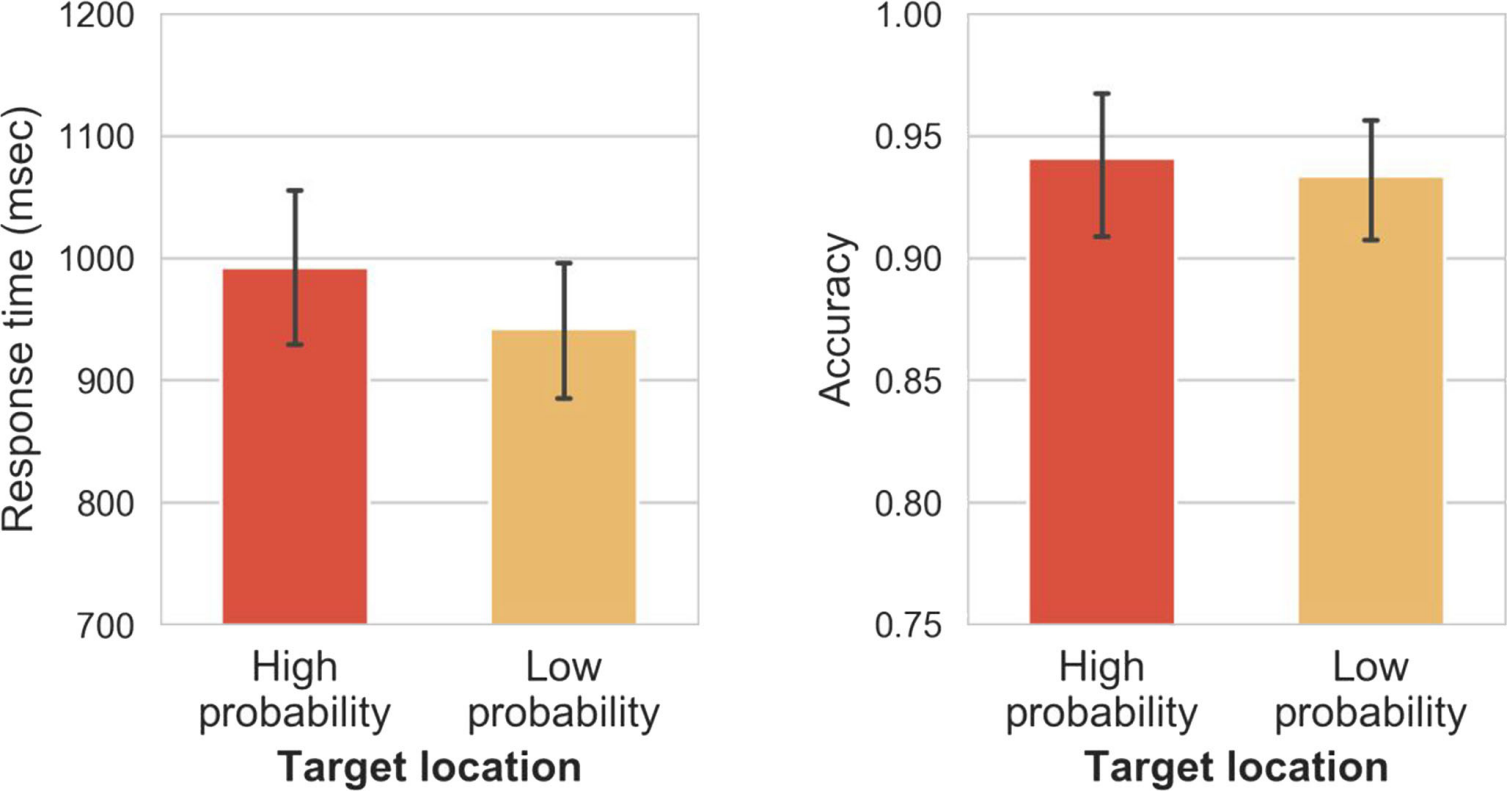
The mean response times (RTs; left panel) and mean accuracy (right panel) between different target conditions (in the absence of a distractor) in Experiment 1. Error bars represent 95% confidence interval (CI)

We also assessed whether the statistical regularities present in the display had an effect on target selection when the target happened to be presented at the high-probability location. For the no-distractor condition, we analyzed mean RTs (see Fig. 3, left panel) with a one-way RM-ANOVA with target location (high-probability vs. low-probability) as a factor. The effect of target location was significant, with target selection being 57-ms slower at the high-probability versus the low-probability locations, *t*(46) = 4.15, *p*_*bonf.*_ < .001, *d =* 0.61, showing that this location was spatially suppressed even when a target singleton was presented there, consistent with Wang and Theeuwes (2018a, b). There was no reliable effect on accuracy, *F*(1, 46) = 0.08, *p*_*bonf.*_ = .77, η_p_^2^ *=* .002 (see Fig. 3, right panel).

#### Distractor suppression and congruence

For these analyses, only distractor-present trials were included, as there was no congruence manipulation in the no-distractor condition.

**Fig. 4:**
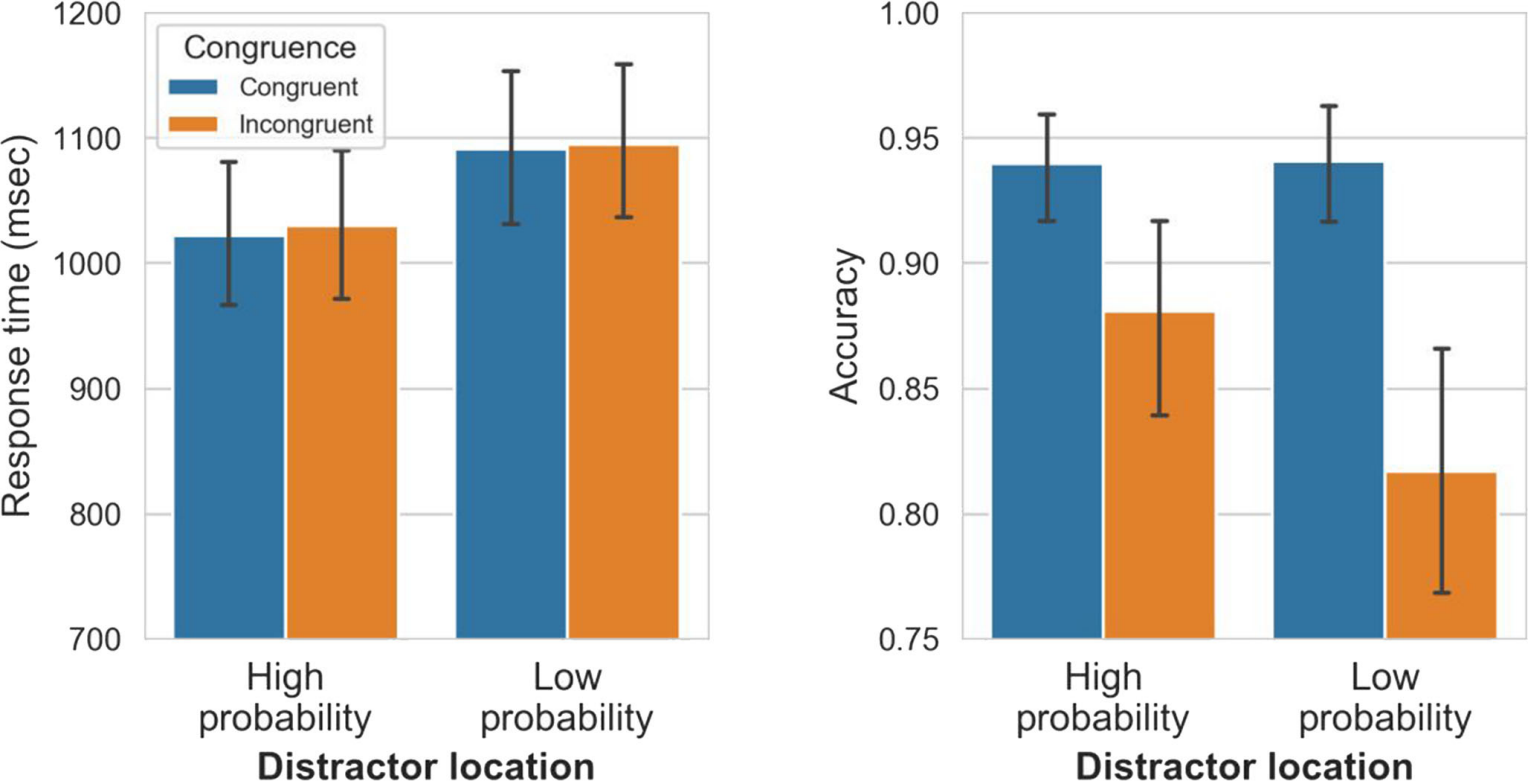
Mean response times (RTs; left panel) and mean accuracy (right panel) between different distractor conditions and different congruence conditions in Experiment 1. Error bars represent 95% confidence interval (CI)

Mean RTs between locations and congruence conditions are shown in Fig. 4 (left panel), and were analyzed using a two-way RM-ANOVA with distractor location (high-probability vs. low-probability) and congruence (congruent vs. incongruent) as factors. Distractor location had a significant main effect on RTs as participants responded 67 ms faster to the target when the distractor was presented at the high-probability relative to the low-probability location, *t*(46) = 8.45, *p*_*bonf.*_ *<* .001, *d =* 1.23. Congruence also had a significant main effect as on average participants responded on average 15-ms faster when the distractor was congruent with the target than when it was incongruent, *t*(46) = 2.20, *p*_*bonf.*_ *=* .03, *d =* 0.32. There was no interaction between distractor location and congruence, *F*(1, 46) = 0.01, *p =* .91, η_p_^2^ *<* .001.

Mean accuracy for locations and congruence conditions is shown in Fig. 4 (right panel), analyzed using a two-way RM-ANOVA, with distractor location (high-probability vs. low probability) and congruence (congruent vs. incongruent) as factors. Distractor location had a main effect: When the distractor appeared at the high-probability location participants were on average 3% more accurate than when the distractor appeared at low-probability locations, *t*(46) = 4.81, *p*_*bonf.*_ *<* .001, *d* = 0.70. Congruence also had a significant main effect, with participants making on average 9% more accurate responses in the congruent than in the incongruent condition, *t*(46) = 5.43, *p*_*bonf.*_ *<* .001, *d* = 0.79. The two-way interaction between distractor location and congruence was significant, *F*(1, 46) = 32.43, *p <* .001, η_p_^2^ *=* .41. Post hoc comparisons show that there was a strong congruence effect within the low-probability locations (i.e., 12.4% lower accuracy in the incongruent vs. congruent condition), *t*(46) = 6.99, *p*_*bonf.*_ *<* .001. Critically, however, the congruence effect in high-probability locations (i.e., 5.8% lower accuracy in the incongruent vs. congruent condition), *t*(46) = 3.26, *p*_*bonf.*_ *=* .01, was smaller than in low-probability locations, *t*(46) = 7.39, *p*_*bonf.*_ *<* .001.

### Discussion

We replicated the main findings of Wang and Theeuwes (2018a, b): reduced capture for when the color distractor was presented at the high-probability location relative to the low-probability location, and less efficient selection of the target when it was presented at the high-probability location (for studies that do not show a similar target suppression, see Sauter, Liesefeld, Zehetleitner, & Müller, 2018; van Moorselaar, Daneshtalab, and Slagter, 2020; Zhang, Allenmark, Liesefeld, Shi, & Müller, 2019). This demonstrates that the results are robust and easily reproduced using an online version of this experiment. In addition to this finding, we found that there was a congruence effect on both RTs and accuracy such that participants took longer and pressed the wrong key more often when the filled side within the distractor was incongruent with the filled side within the target. In other words, if the color distractor contained information that suggested the opposite response than the response that was required during that trial (the response afforded by the target), participants made more errors consistent and reminiscent of the classic flanker effect (e.g., B. A. Eriksen & Eriksen, 1974). Critically however, for accuracy, this congruence effect was very much attenuated when the distractor singleton was presented at a high-probability location suggesting that this location was suppressed relative to all other locations. However, the reduction of interference from the suppressed location was not perfect, suggesting that even when presented at the high-probability location the distractor still captured attention enough to cause response interference.

Our findings suggest that suppression was proactive, as the congruence effect for the high-probability location was very much reduced relative to the low-probability locations. If anything, congruence effects were not reversed, which would have indicated reactive suppression. Even though the high-probability location was proactively suppressed, this suppression was far from perfect, as there was still a reliable attentional capture effect, as well as a congruence effect for the high-probability location. This finding is consistent with an eye-tracking study using the same paradigm (Wang, Samara & Theeuwes, 2019). This study showed that in the majority of trials, there was suppression of the high-probability location resulting in fewer eye movements going to that location. However, on a subset of trials the eyes did go to the high-probability location; in those cases, the eyes moved away quicker from the high-probability location than from low-probability locations, suggesting that attention can be disengaged is faster from the high-probability location than from the low-probability locations. Because disengagement is faster at the suppressed location, it may result in a reduced congruence effect from distractors appearing there.

## Experiment 2

In Experiment 2, we added squares and triangles as display elements such that the target singleton had no longer a unique shape. This manipulation ensured that the target shape would not “pop-out” from the display. In addition, the target was always a circle making it possible to consistently search for the same shape throughout the experiment. This forces participants to adopt the so-called feature-search mode (Bacon & Egeth, 1994; Leber & Egeth, 2006), which is assumed to impose top-down control that should eliminate capture by stimuli that do not match the attentional set (e.g., Leber & Egeth, 2006). Specifically, due to this “feature-search” top-down set, participants should be able to completely ignore the salient distractor (Bacon & Egeth, 1994; Leber & Egeth, 2006; Theeuwes, 2010).

Recently, however, it has been argued that when engaged in feature-search, it is not merely that the salient distractor singleton is ignored, but instead is actively suppressed below baseline (i.e., “signal-suppression hypothesis”; Gaspelin, Leonard, & Luck, 2015; Gaspelin & Luck, 2018a, 2018b), which should prevent attentional capture. This is confirmed by studies showing that when engaged in feature-search (but not in the singleton-detection mode), the irrelevant distractor singleton elicits a PD component of the event-related potential (ERP) signal, which is assumed to be a neural maker of suppression (Feldmann-Wüstefeld, Uengoer, & Schubö, 2015; Hickey, Di Lollo, & McDonald, 2008; Sawaki & Luck, 2013).

Therefore, by engaging participants in feature-search mode, we expect suppression of the distractor singleton regardless of whether it is presented at a high-probability or a low-probability location. Therefore, due to the suppression of the distractor singleton, we expect no congruence effect, as attention is never directed to the distractor singleton. It is reasoned that if spatial attention is never directed to the location of the distractor singleton, there cannot be a congruence effect (see also Theeuwes, 1996; Theeuwes, Atchley, & Kramer, 2000; but see Folk & Remington, 2006, for a different argument). Alternatively, a congruence effect will be observed if the distractor singleton still captures attention.

### Method

The study was approved by the Ethical Review Committee of the Faculty of Behavioral and Movement Sciences of Vrije Universiteit Amsterdam.

#### Participants

Participant recruitment was done through Prolific (www.prolific.co). Fifty participants completed the task online (21 females; *M*_age_ = 27.2 years). Power was calculated in the same manner as in Experiment 1, with the only difference that an effect size for distractor location was obtained from Wang and Theeuwes (2018b) where the feature-search mode was also employed. All power analyses suggest we had >.99 power for the critical effects. All participants reported normal to corrected-to-normal (color) vision. Before the experiment, participants provided informed consent via button press.

#### Apparatus and stimuli

**Fig. 5:**
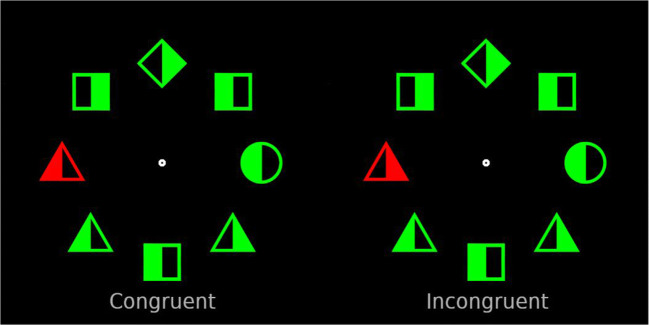
Example search displays from Experiment 2. The search target was always a circle. The display to the left represents the congruent condition; the display to the right represents the incongruent condition. Stimuli are not displayed to size

The experiment design and stimuli were the same as in Experiment 1, except for the addition of two new shapes (see Fig. 5): squares subtended 85 × 85 px; and triangles subtended 100 × 100 px. On each display there were always three squares, three triangles, one diamond, and one circle, with their locations determined randomly on each trial.

#### Procedure and design

The task was identical to Experiment 1, with the exception that the target was a circle on every trial. The shape of the distractor singleton was randomly determined on each trial. The distractor singleton location probabilities were the same as in Experiment 1. Half of the distractor-present trials were congruent with the target circle (i.e., left-left), and the other half were incongruent (i.e., left-right).

### Results

Trials in which response times (RTs) were below or above a 2.5 standard deviation cutoff point (within-participants) were excluded from analyses (2.4% of all trials).

#### Attentional capture and distractor suppression

**Fig. 6:**
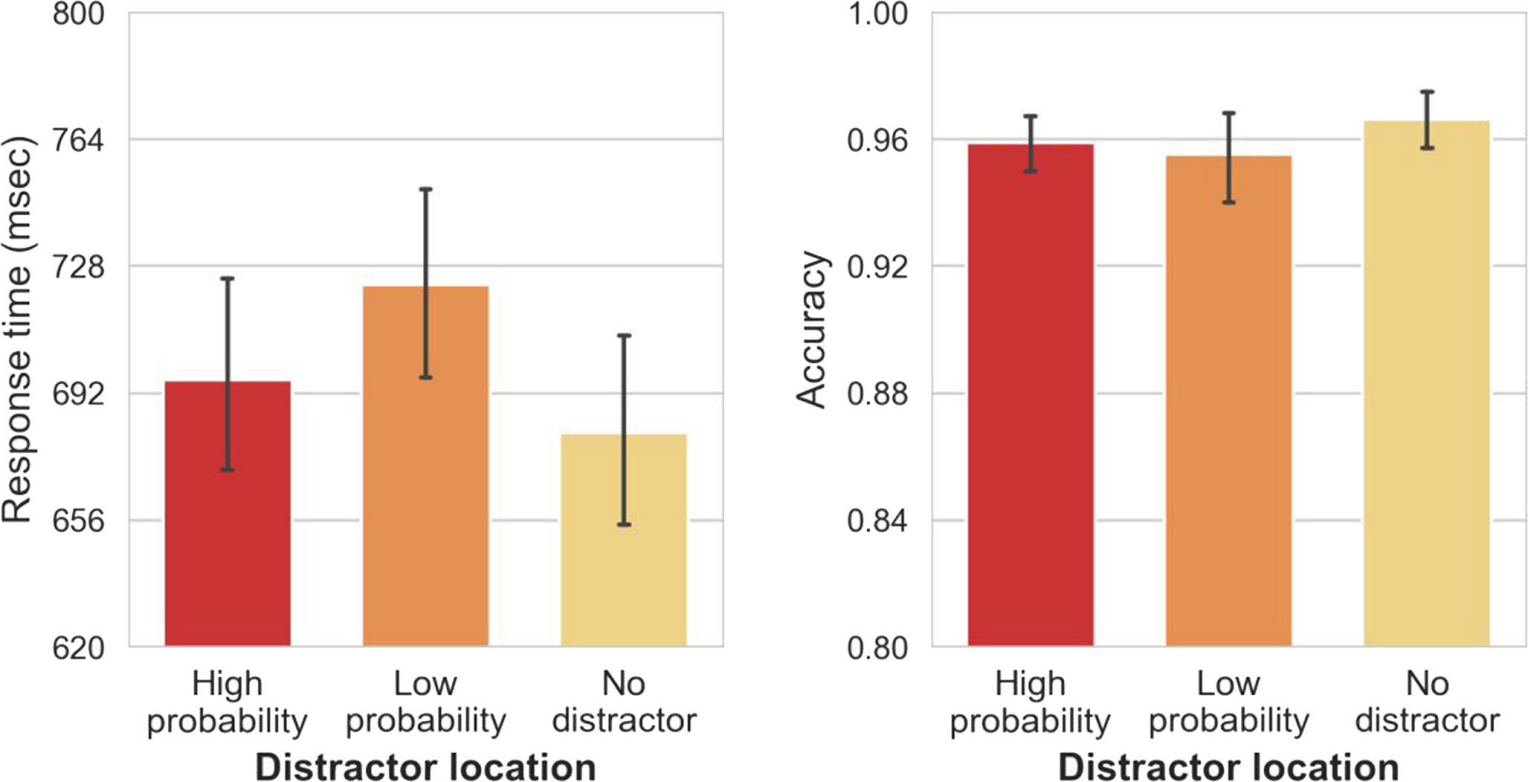
Mean response times (RTs; left panel) and mean accuracy (right panel) between different distractor conditions and different congruence conditions in Experiment 2. Error bars represent 95% confidence interval (CI)

Mean RTs are shown in Fig. 6 (left panel), and were analyzed using a two-way RM-ANOVA, with distractor location (no distractor vs. high-probability vs. low-probability) and repetition (yes vs. no) as factors. Statistics are reported with the Greenhouse–Geisser sphericity correction where necessary. Distractor location had a significant main effect, *F*(1.60, 78.44) = 46.23, *p <* .001, η_p_^2^ *=* .48. Repetitions did not have a main effect, *F*(1, 49) = 3.41, *p =* .07, η_p_^2^ *=* .06. A Bayesian analysis suggested that that the data are 2.96 (BF_01_) more likely under a null model than a model including the main effect of repetition, providing more evidence that the effect is not solely driven by trial-to-trial repetitions. Post hoc comparisons show that responses in the no-distractor condition were on average 40-ms faster than in the low-probability condition, *t*(49) = 9.52, *p*_*bonf.*_ *<* .001, *d =* 1.35, and 15 ms faster than in the high-probability condition, *t*(49) = 3.61, *p*_*bonf.*_ *=* .001, *d =* 0.51. There was suppression of the high-probability location as responses there were 24 ms faster than responses in low-probability locations, *t*(49) = 5.91, *p*_*bonf.*_ *<* .001, *d =* 0.84.

Mean accuracy is shown in Fig. 6 (right panel), and was analyzed using a two-way RM-ANOVA with distractor location (no distractor vs. high-probability vs. low-probability) and repetition (yes vs. no) as factors. Distractor location had a reliable main effect, *F*(1.6, 78.45) = 6.47, *p =* .004, η_p_^2^ *=* .12. Repetitions, however, also had a reliable main effect: when the distractor location condition was the same as in the previous trial, responses were on average 1.3% less accurate than when the conditions changed between trials, *t*(49) = 3.78, *p* < .001, *d* = 0.53. There was no interaction between the two factors, *F*(1.83, 89.73) = 2.29, *p* = .112, η_p_^2^ *=* .04. Post hoc comparisons show that when there was no distractor, participants made on average 1.6% more accurate responses than when the distractor was in a low-probability location, *t*(98) = 3.63, *p*_*bonf.*_ = .001, *d* = 0.51. However, there was no difference in accuracy between the no-distractor and high-probability conditions (0.6% mean difference), *t*(98) = 1.33, *p*_*bonf.*_ *=* .56, *d* = 0.19, suggesting that response selection was not at all affected when distractors appeared in the high-probability location.

**Fig. 7:**
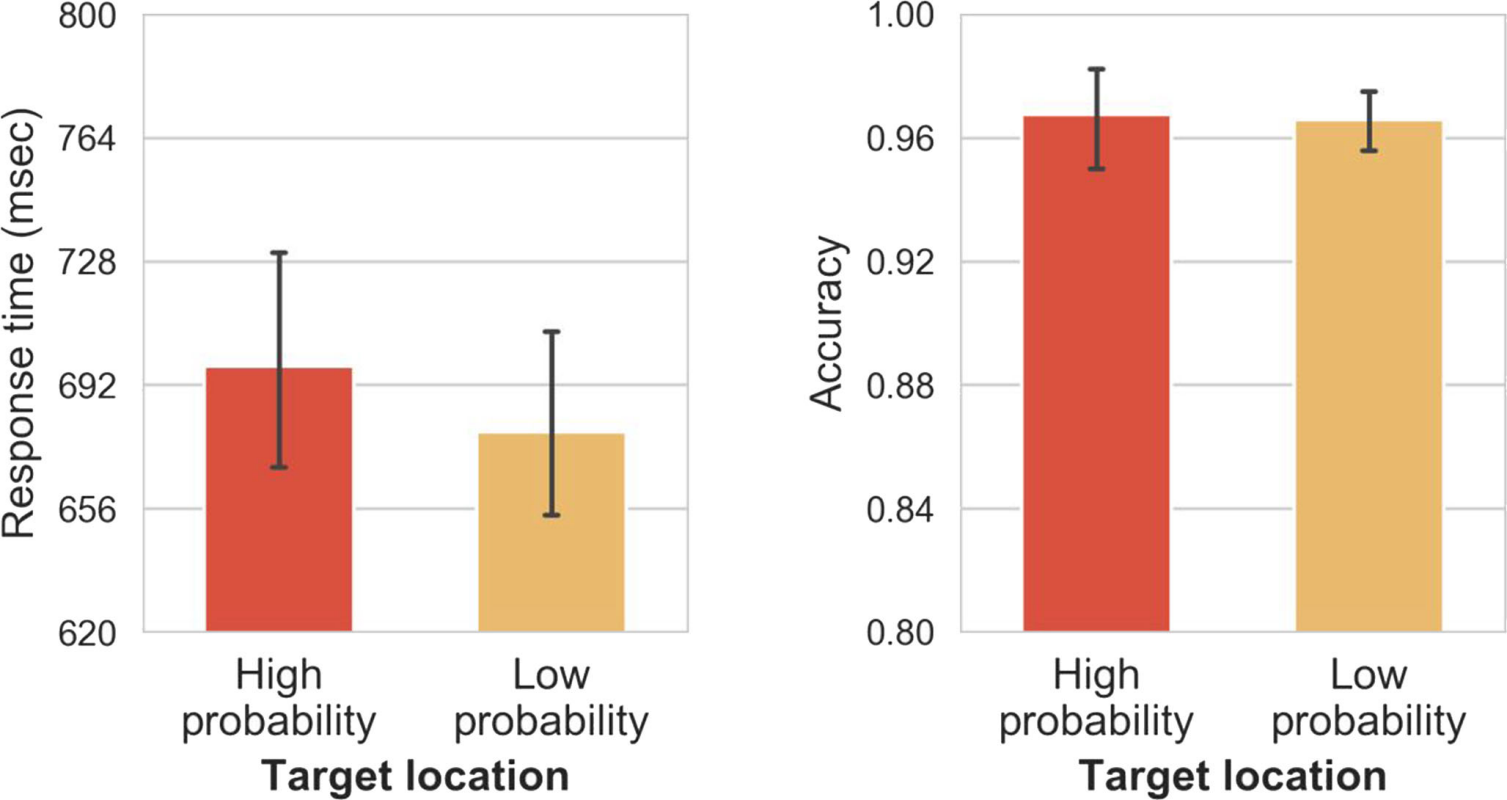
Mean response times (RTs; left panel) and mean accuracy (right panel) between different target conditions in the absence of a distractor in Experiment 2. Error bars represent 95% confidence interval (CI)

We again assessed whether the statistical regularities present in the display had an influence on target selection even in the no-distractor condition. We analyzed RTs (see Fig. 7, left panel) with a one-way RM-ANOVA, with target location (high-probability vs. low-probability) as a factor. Even with the reduced levels of attentional capture in this experiment, there was an effect of target location on RTs: participants selected the target 23 ms slower when it was presented at the high-probability versus the low-probability locations, *t*(49) = 3.02, *p*_*bonf.*_ *=* .004, *d =* 0.43. There were no reliable effects on accuracy, *F*(1, 49) = 0.01, *p =* .90, η_p_^2^ *<* 0.001 (see Fig. 7, right panel).

#### Distractor suppression and congruence

For these analyses, only distractor-present trials were included, as there was no congruence manipulation in the no-distractor condition.

**Fig. 8:**
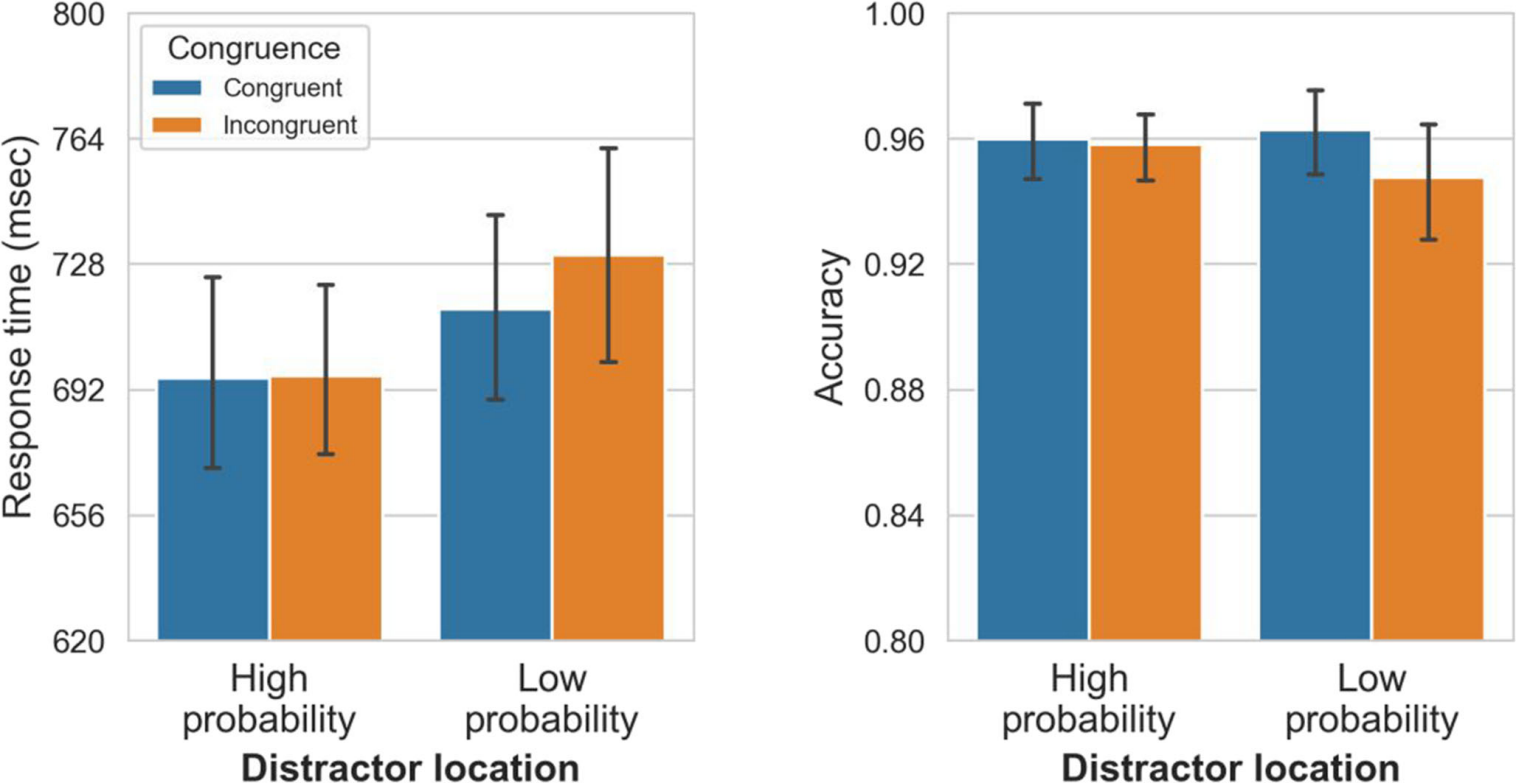
Mean response times (RTs; left panel) and mean accuracy (right panel) between different distractor conditions and different congruence conditions in Experiment 2. Error bars represent 95% confidence interval (CI)

Mean RTs between locations and congruence conditions are shown in Fig. 8 (left panel), and were analyzed using a two-way RM-ANOVA, with distractor location (high-probability vs. low-probability) and congruence (congruent vs. incongruent) as factors. Distractor location had a significant main effect on RTs: participants responded on average 27-ms faster when the distractor was at the high-probability location than when it was in the low-probability locations, *t*(49) = 7.86, *p*_*bonf.*_ < .001, *d =* 1.11. Congruence also had a reliable main effect: participants responded on average 8-ms faster in congruent conditions than in incongruent conditions, *t*(49) = 3.11, *p*_*bonf.*_ = .003, *d* = 0.44. There was also a significant interaction between distractor location and congruence, *F*(1, 49) = 6.61, *p =* .01, η_p_^2^ *=* .12. Crucially, for the high-probability location there was no difference in RTs between congruence conditions (i.e., 0.6 ms mean difference; *t* < 1); whereas in the low-probability location, incongruent conditions resulted in slower responses compared to congruent conditions (i.e., 14-ms mean difference), *t*(49) = 4.02, *p*_*bonf.*_ *<* .001.

Mean accuracy between locations and congruence conditions is shown in Fig. 8 (right panel), and was analyzed using a two-way RM-ANOVA, with distractor location (high-probability vs. low-probability) and congruence (congruent vs. incongruent) as factors. Distractor location did not have a reliable main effect, *F*(1, 49) = 0.83, *p =* .37, η_p_^2^ *=* .02. Congruence had a significant main effect: congruent conditions led to 0.09% more accurate responses on average than Incongruent conditions, *t*(49) = 2.09, *p*_*bonf.*_ *=* .041, *d* = 0.27. There was no interaction between distractor location and congruence, *F*(1, 49) = 2.60, *p* = .11.

### Discussion

The current study shows that even when participants search for a specific feature (a circle between squares and triangles), the distractor singleton still captured attention. This is consistent with previous studies showing that even when participants have to engage in feature-search mode, a salient irrelevant singleton can capture attention (Graves & Egeth, 2016; Theeuwes, 2004; Vatterott & Vecera, 2012; Wang & Theeuwes, 2018b; Zehetleitner, Goschy, & Müller, 2012). Recently, Wang and Theeuwes (2020b) shed some light on this issue by showing that when engaged in feature-search, only in displays consisting of a few heterogeneous items, participants are able to ignore the distractor singleton (see also, Theeuwes, 2004). If the target and distractor are salient enough (in displays with enough items), regardless of the search mode employed, there is capture by the irrelevant distractor singleton. In the current experiment, with eight items on display, the distractor was salient enough to capture attention even when participants had to search for a specific feature (i.e., feature-search mode).

Also, and consistent with Wang and Theeuwes (2018b), even when using feature-search, there is still a reliable effect of the statistical regularity as capture was smaller in the high-probability relative to the low-probability location. This indicates that even in feature-search mode, in which participants are assumed to completely ignore the irrelevant distractor (Bacon & Egeth, 1994), participants learn the statistical regularity regarding this salient distractor, affecting both the amount of attentional capture by the distractor and the efficiency of selection of the target. Again, this indicates that the salient distractor was not ignored even when using the feature-search mode.

The analysis of the congruence effect also suggests that the distractor singletons captured attention: participants made more errors and responded slower in the incongruent versus the congruent condition, but only in low-probability conditions. For the high-probability location, there was no reliable congruence effect on RTs or accuracy suggesting proactive suppression of the high-probability location. At low-probability locations, participants responded slower and less accurately when the distractor was incongruent with the target than when it was congruent, although the difference in accuracy was not statistically significant.

We conclude that even in feature-search mode, the high-probability location was suppressed proactively as there was no congruence effect anymore; yet even though response interference from distractors appearing at that location was abolished, they were not suppressed below baseline, as attentional capture was not completely eliminated. Indeed, when a distractor was present in the high-probability location, RTs were about 15-ms slower than when there was no distractor. This suggest that nonspatial filtering costs may also play a role (Kahneman, Treisman, & Burkell, 1983). These costs are assumed to reflect slower allocation of attention to the target singleton because distracting stimuli need to be filtered out of the competition for attentional resources with the target. However, according to the nonspatial filtering hypothesis, there is no shift of spatial attention to the location of the distractor singleton (see also Schreij, Owens, & Theeuwes, 2008).

## General discussion

Our results are a successful replication of Wang and Theeuwes (2018a, 2018b) in an online sample of participants, showing evidence that locations having a high probability of containing a salient distractor are suppressed relative to other locations in a visual search array. We show that the capture by a salient distractor was reduced (Experiment 1) and nearly eliminated (Experiment 2) when this salient distractor was presented at a high-probability versus a low-probability location. Furthermore, when the target happened to be presented at the high-probability location, selection was less efficient than when the target was presented at the low-probability location. This suggests that the suppression of the high-probability location is feature-blind, as both distractors and targets are suppressed alike.

To further test the extent to which the high-probability location is suppressed, we introduced “flanker” distractors (e.g., B. A. Eriksen & Eriksen, 1974; Wei, Kang, & Zhou, 2013), that could either be congruent or incongruent with the target response. We observed congruence effects in both experiments: Incongruent distractors led to more errors or slowed down responses, compared with congruent distractors. In Experiment 1, there was a congruence effect for both high-probability and low-probability locations, suggesting that in both conditions, attention was captured towards the location having a salient distractor. However, the congruence effect was smaller in the high-probability relative to the low-probability location, suggesting that at least on a subset of trials, the high-probability location was suppressed successfully. In Experiment 2 there was no congruence effect for the high-probability location, suggesting that attention never went there. This latter finding suggests proactive suppression of that location. Critically, there was a clear congruence effect for the low-probability location even though participants had to use the feature-search mode to find the target.

It is unclear why in Experiment 1 the congruence manipulation mainly affected accuracy, while in Experiment 2 the effect was mainly found for RT. Clearly, in Experiment 1 capture was much stronger than in Experiment 2, which may result in attention dwelling longer at the distractor location than in Experiment 1. Because attention dwells longer, the response associated with the orientation of the distractor gets activated, which may ultimately lead to executing that response. If it happens to be congruent with the target, the response that is committed is correct; however, if it is incongruent, participants will commit an erroneous response. In Experiment 2, overall capture was reduced. As argued, because the high-probability location was proactively suppressed, the orientation of the distractor was not processed, and therefore there was no congruence effect. For the low-probability locations, attention was captured such that the orientation of the distractor was processed, giving rise to slower responses when distractor and target were incongruent.

One aspect that is important to highlight is that in Experiment 2, participants had to use the *feature-search mode* to find a specific shape (in our case, a circle) among a heterogeneous set of distractor shapes. It is generally agreed that when the feature-search mode is used, top-down control should prevent attentional capture by the salient singleton (Bacon & Egeth, 1994; because “subjects using this mode should not be susceptible to capture by stimuli not matching the attentional set”; Leber & Egeth, 2006, p. 133). If indeed feature-search makes it possible to completely ignore the distractors we should not find a congruence effect. Our results show that for the low-probability location there is a clear congruence effect suggesting that the distractors did capture attention. When the distractor is presented at the high-probability location, proactive suppression prevents capture and the processing of the distractor orientation. Because the distractor orientation is not processed there cannot be a congruence effect.

An alternative account for what is used in the literature as the feature-search mode was recently provided by Wang and Theeuwes (2020a, b; see also Theeuwes, 2004), who argued that by increasing the number of elements having unique features in the display (which is typically done to induce feature-search), the salience of both the target and the distractor stimuli is reduced. This forces observers to employ a more serial search strategy, which in turn reduces (or eliminates) attentional capture. There is no need to assume any top-down filtering. Our data support this notion: the distractor singleton clearly captured attention in Experiment 2, but in magnitude the capture in Experiment 2 was much smaller than in Experiment 1.

In sum, regardless of the search mode employed, the location that is most likely to contain a distractor singleton is proactively suppressed such that this location competes less for attention within the spatial priority map than in all other locations.

### Open practices statement

None of the data or materials for the experiments reported here is currently available, but will be provided upon request. None of the experiments was preregistered.
